# Inflammatory Bowel Disease: New Insights into the Interplay between Environmental Factors and PPARγ

**DOI:** 10.3390/ijms22030985

**Published:** 2021-01-20

**Authors:** Giulia Caioni, Angelo Viscido, Michele d’Angelo, Gloria Panella, Vanessa Castelli, Carmine Merola, Giuseppe Frieri, Giovanni Latella, Annamaria Cimini, Elisabetta Benedetti

**Affiliations:** 1 Department of Life, Health and Environmental Sciences, University of L’Aquila, 67100 L’Aquila, Italy; giulia.caioni@guest.univaq.it (G.C.); angelo.viscido@univaq.it (A.V.); michele.dangelo@univaq.it (M.d.); gloria.panella86@gmail.com (G.P.); vanessa.castelli@univaq.it (V.C.); giuseppe.frieri@univaq.it (G.F.); giovanni.latella@univaq.it (G.L.); annamaria.cimini@univaq.it (A.C.); 2 Faculty of Bioscience and Technology for Food, Agriculture and Environment, University of Teramo, Via Balzarini 1, 64100 Teramo, Italy; cmerola@unite.it; 3 Sbarro Institute for Cancer Research and Molecular Medicine and Center for Biotechnology, Temple University, Philadelphia, PA 19122, USA

**Keywords:** PPARγ, pathophysiological processes of IBD, environmental factors, IBD models

## Abstract

The pathophysiological processes of inflammatory bowel diseases (IBDs), i.e., Crohn’s disease (CD) and ulcerative colitis (UC), are still not completely understood. The exact etiology remains unknown, but it is well established that the pathogenesis of the inflammatory lesions is due to a dysregulation of the gut immune system resulting in over-production of pro-inflammatory cytokines. Increasing evidence underlines the involvement of both environmental and genetic factors. Regarding the environment, the microbiota seems to play a crucial role. Peroxisome proliferator-activated receptors (PPARs) are nuclear receptors that exert pleiotropic effects on glucose homeostasis, lipid metabolism, inflammatory/immune processes, cell proliferation, and fibrosis. Furthermore, PPARs modulate interactions with several environmental factors, including microbiota. A significantly impaired PPARγ expression was observed in UC patients’ colonic epithelial cells, suggesting that the disruption of PPARγ signaling may represent a critical step of the IBD pathogenesis. This paper will focus on the role of PPARγ in the interaction between environmental factors and IBD, and it will analyze the most suitable in vitro and in vivo models available to better study these relationships.

## 1. Introduction

The worldwide epidemiology of inflammatory bowel diseases (IBDs), such as Crohn’s disease (CD) and ulcerative colitis (UC), has been influenced mainly by industrial progress and the improvement of human living conditions [1]. The increasing incidence and prevalence of IBD in developing countries suggest a connection with a westernized lifestyle and changed habits. The etiopathogenesis of IBD is not entirely understood. However, it is hypothesized to be related to a mixture of factors, including genetic susceptibility, dysregulation of the gut immune system, and environmental elements in conjunction with the microbiota. There is a need for complete information about these diseases since they represent an expanding global health problem, the costs of which are challenging to manage.

CD and UC are chronic inflammatory disorders with distinct clinical characteristics. CD can affect all gastrointestinal tract segments (most commonly the terminal ileum and colon) in a non-continuous manner, causing a typically asymmetrical, segmental, and transmural inflammation. CD complications include abscesses, fistulas, and strictures, and many patients need surgical procedures. UC involves the colonic mucosal surface, primarily affecting the rectum and, in some cases, the entire colon in a continuous manner. Depending on the extent of inflammation, UC can evolve into several forms, from proctitis to left-sides colitis or pancolitis [2]. Behind the different characteristics, CD and UC share almost common features regarding risk factors, symptoms, clinical course, complications, and the absence of a definitive cure. The current therapeutic approaches aim to reduce intestinal inflammation and, more specifically, block pro-inflammatory cytokines. However, the conventional therapy of IBD, i.e., salicylates, steroids, and immunosuppressants reduce the mortality, but not the rate of complications and surgery; the latter is required in up to 70% of CD and 20% of UC patients during their lifetime. The introduction of biological therapies, i.e., anti-tumor necrosis factor-alpha (TNF-α), anti-α4β7 integrin, and anti-interleukins 12/23 antibodies, caused a revolution in the treatment of IBD; however, they reduced hospitalization, complications, and surgery only in the short- and medium-term. For these reasons, it is necessary to find personalized treatments employing new strategies for drug monitoring and, above all, identifying useful targets.

Concerning the underlying mechanism, IBD’s pathogenesis seems to be related to an alteration in the innate immune system, including epithelial barrier defects with changes in E-cadherin, β-catenin, and claudins expression [3] and an inadequate expression of antimicrobial peptides. The gut immune homeostasis can be disrupted by innate immune cells contribution (neutrophils, dendritic cells, and macrophages) and the release of inflammatory mediators [4]. IBDs are typically characterized by high levels of cytokines, such as TNF-α, IL-6, IL-8 (one of the first chemokines described), IL-12, and chemokines such as chemokine ligand 2, chemokine ligand 3, and chemokine ligand 1 in colon tissues [5]. However, the role of adaptive immunity cannot be omitted since, in CD and UC patients, an alteration in immunoglobulin subclass production has been found [6]. Among other pathogenic components, there is an impairment in Peroxisome proliferator-activated receptors-γ (PPARγ) activity, abnormalities of the enteric nervous system, genetic variants, and the presence of regulatory RNAs [4]. The high expression of PPARγ in the bowel has already been demonstrated [7], and several studies have shown its role in human colonic inflammation [8] along with the involvement of immune system response [9]. Notably, mesalazine, the most used drug in UC, binds and activates PPARγ [10]. Specifically, mesalazine enhances PPARγ expression and promotes its translocation from the cytoplasm to the nucleus [10]. Different xenobiotics and environmental pollutants can influence and alter the Peroxisome proliferator-activated receptors (PPAR) signaling pathway [11]. This evidence suggests a relationship between external factors and the onset of gut inflammation diseases.

This review analyzes and discusses the complex interaction of environment and PPARγ mediated-pathways as part of IBDs’ pathogenesis. It will describe the current suitable in vitro and in vivo models with particular attention to each one’s advantages and disadvantages. It will contribute towards a better understanding of PPARγ biology to expand the effective treatment strategies and improve patients’ conditions.

## 2. PPARs: Crosstalk between Metabolism and Inflammation

PPARs are ligand-dependent transcription factors and belong to nuclear hormone receptors’ superfamily, playing an important role in lipid and glucose metabolism [12]. In mammals, three isoforms of PPARs have been identified: PPARα or NR1C1, PPARβ/δ or NR1C2, and PPARγ or NR1C, each of them encoded by a different gene. They share a similar structure. The ability to bind agonists is mediated by a ligand-binding domain (LBD) in the C-terminus, while the DNA binding domain is in the N-terminus. These receptors can be activated by natural fatty acids and eicosanoids or synthetic ligands, which are used in the clinical management of metabolic diseases, such as fibrates, with cardioprotective properties [13], and thiazolidinediones, used in the treatment of diabetes mellitus type 2 [14]. After interaction with agonists, they are translocated in the nucleus, and their function depends on the heterodimerization with retinoid X receptor (RXR). The heterodimers bind to sequence-specific PPAR response elements (PPREs), stimulating the target genes’ transcription [15].

The members of the PPARs family show a wide range of actions on glucose and lipidic homeostasis, and they share many similarities in terms of structure and function; however, each isoform has a specific physiological activity, influenced by their tissue distribution.

PPARα is expressed in tissues that require a large amount of energy, principally the liver, kidney, and skeletal muscle; its localization has also been demonstrated in cardiomyocytes, intestinal mucosa, adrenal gland, brown adipose tissue, and brain [16,17]. It is involved in the catabolism of fatty acids and their oxidation [18]. Regarding PPARβ/δ, this isoform is involved in several processes, including cell proliferation, differentiation, migration, and apoptosis. Its activity is also related to glucose and cholesterol homeostasis, insulin sensitivity, and angiogenesis [17,19]. It is ubiquitously expressed but particularly abundant in the gastrointestinal tract, kidneys, skeletal muscle, and brain [20]. PPARγ is abundantly expressed in white and brown adipose tissue, where it plays a crucial role in regulating adipogenesis, energy balance, and lipid biosynthesis. It is also expressed in the intestines, liver, kidneys, brain, immunological system, and muscles [17,21].

PPARs are usually described as the main actors in lipid and glucose metabolism, but much evidence indicates their involvement in controlling inflammatory responses and inflammation-related disorders such as fibrosis and cancer. In addition to their known anti-inflammatory action, PPARs also modulate fibrogenesis and carcinogenesis.

It has been reported that PPARs have anti-inflammatory potential, modulating several points of inflammatory pathways. Inflammation consists of a dynamic sequence of phenomena, including the release of mediators that leads to vasodilatation, increased blood flow, vascular permeability, and recruitment of polymorphonuclear cells, particularly neutrophils in acute phase of inflammatory process, whereas mononuclear cells, macrophages, T- and B-lymphocytes are in chronic immunomediated inflammation. PPARs could intervene at each level of these processes. For example, PPARα can negatively interfere with the NF-κB signaling pathway, repressing several inflammatory genes such as VCAM-1, COX-2, and IL-6 [22]. PPARα is also involved in inhibiting the expression of inducible nitric oxide synthase [23] and TNF-α in macrophages [24]. PPARβ/δ activity is induced in the host inflammatory response in the skin, and it results in being up-regulated in keratinocytes as a consequence of external triggers. The activation of PPAR-β/δ-pathway determines the expression of genes related to keratinocyte differentiation, survival, and repair [25]. Other studies focused on the role of PPARβ/δ in attenuating atherosclerosis progression, revealing that this isoform has an HDL-raising effect and anti-inflammatory activity within the vessel wall, where it participates in the down-regulation of chemokines production [26].

Mechanisms of the anti-inflammatory effects of PPARγ include the inhibition of the transcriptional activity of NF-κB, STAT-1, and AP-1 [27]. A direct relationship between TNF-α adipocyte secretion and a decrease in expression of PPARγ has been reported [28]. Moreover, negative regulation of PPARγ contributes to the antiadipogenic effects of TNF-α, whose increased production is relevant in obesity states [29]. In fact, several studies have also demonstrated a metabolic benefit related to the anti-inflammatory effects of targeting PPARγ [30].

All PPARs isoforms can have a role in regulating inflammatory responses, employing their interaction with various transcription factors stimulating inflammation, signal transducer, the formation of complexes between co-activators and co-repressors, and the modulation of different kinases [31].

Thus, the need for more in-depth knowledge of PPARs activity derives from their key role in various metabolic processes, including lipid and glucose homeostasis and inflammatory disease, which makes them the ideal target for developing new pharmacological strategies.

PPAR agonists are currently used to treat many diseases, such as hyperlipidemia, insulin resistance, type 2 diabetes, cardiometabolic syndrome, and atherosclerosis. The latest generation of agonists is represented by the selective peroxisome proliferator-activated receptor modulators, indicated as SPPARMs. With respect to traditional ligands, they can act as dual partial agonists, binding to two isoforms of receptors. Moreover, they have the ability to produce particular conformational changes, which leads to the preferential activation of transcriptional factors [32]. Further progress is represented by pan agonists, whose beneficial effects result from the ability to bind and activate the diverse isoforms of PPARs, reinforcing the single activation mediated by selective agonists [33].

### The Importance of PPARγ in IBDs

Maintaining a healthy gastrointestinal tract depends on external factors (diet, chemicals, drugs, stress) or endogenous factors (genetics, microbiota, efficient immune responses). The loss of equilibrium between these factors may determine the onset of diseases. However, the elements contributing to inflammation and bowel disease are not clear. It is not possible to define a single factor or gene responsible for these modifications. Considering the critical role of PPARs in inflammation, many studies have focused on the possible correlation between these receptors, IBD, and cancer. In particular, chronic intestinal inflammation represents the leading risk factor for the development of gastrointestinal malignancy. Patients with UC and CD have a significantly higher cancer susceptibility [34].

The role of PPARs in inflammatory responses, which has already been described above, makes them possible actors in the pathogenesis of intestinal disorders and cancer. Particular attention has been given to PPARγ since it is abundantly expressed in epithelial cells in the large and small intestines [7]. Although the first evidence on the potential link between PPARγ and intestinal disease established a correlation between this receptor expression and an increase in colon tumorigenesis, recently, many researchers have re-examined the association between PPARγ and the risk of colorectal cancer (CRC) [35,36,37,38].

Genetic studies have demonstrated that heterozygous intestinal-specific PPARγ knockout enhanced tumor growth, evidencing PPARγ as a tumor resistance factor [39]

Interestingly, the activation of PPARγ by mesalazine could be responsible for CRC prevention observed with this drug in IBDs [40]. In immune-deficient mice engrafted with human CRC cells, mesalazine administration reduces xenografts’ growth via a PPAR-γ-dependent mechanism [41]. Activation of PPARγ by mesalazine is accompanied by induction of the tumor suppressor gene PTEN, activation of caspase-8 and caspase-3, and diminished expression of surviving and X-linked inhibitor apoptosis protein [42].

However, the role of PPARγ is not only related to tumorigenesis, because a lot of evidence suggests its involvement in inflammation diseases. For example, a down-regulation has been reported in PPARγ expression in UC [37], and a negative correlation has also been hypothesized with UC progression, because of the low level of PPARγ mRNA in the mucosa of active UC patients compared with UC patients in remission [8]. Sugawara et al. demonstrated positive evidence for the association of allelic variation of the PPARγ gene and CD [43]. These findings suggest that chronic inflammation could be caused by decreased levels in PPARγ expression in the colon.

Among the distinctive features of IBDs’ pathogenesis, there is real deregulation in cytokine production in inflamed colon areas. Studies on human biopsies and in vitro models have demonstrated that a broad set of molecules dominates the mucosal response, where the contribution of epithelial cells is predominant [44]. These soluble mediators include pro-inflammatory cytokines, such as TNF-α, IFN-γ, IL-6, IL-12, IL-21, IL-23, IL-17, and anti-inflammatory cytokines, such as IL-10, TGF*β*, IL-35 [4] and chemokines CXCL1, CXCL2, CXCL3, CCL20 [44]. In particular, the elevated production of IL-12, IL-23, IFN-*γ*, and IL-17 seems to be characteristic of CD, while UC is usually associated with increased production of IL-3, IL-5, and IL-9 [5].

**Figure 1:**
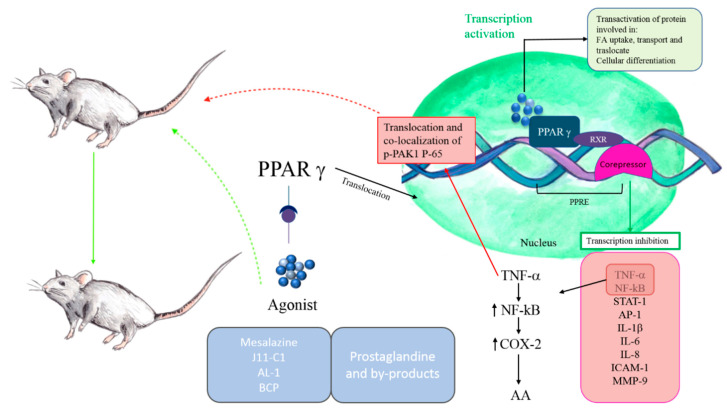
Peroxisome proliferator-activated receptors-γ (PPARγ) in IBDs. PPARγ belongs to the family of nuclear receptors. Its activation involves the translocation in nucleus and the heterodimerization with retinoid X receptor (RXR). The heterodimers bind to sequence-specific PPAR response elements (PPREs), stimulating the transcription of target genes. Corepressors maintain the target genes inactivated in absence of PPARγ ligands. The protective effects of PPARγ include the modulation of pro-inflammatory cytokines production, such as TNFγ, IL-6, the inhibition of transcription factors, including NF-kB, STAT-1, AP-1, and intercellular adhesion molecule and MMP-9. PPARγ also determines the downregulation of p65 expression and IkappaB kinase. In contrast, TNF-α activates NF-kB, which stimulates COX-2 to convert arachidonic acid in prostaglandins. The anti-inflammatory properties of prostaglandins are related to their ability to bind PPARγ, blocking NF-kB downstream events. PPARγ synthetic agonists can ameliorate IBD inflammation.

PPARγ distinguishes itself for the protective effects, including the modulation of cytokine/chemokine production and the negative regulation of macrophage activation [45]. In this scenario, it is not difficult to understand why studies have abundantly focused on the γ-isoform of PPARs and the possible mechanism through which this receptor could be involved in bowel inflammation (Figure 1).

The activation of PPARγ determines a decrease in the production of pro-inflammatory cytokines, such as TNF-α and IL-6, and the inhibition of transcription factors, including NF-κB, AP-1, STAT-1, and Intercellular Adhesion Molecule (ICAM-1), and matrix metallopeptidase 9 (MMP-9) [46].

Several studies have shown that PPARγ synthetic agonists can ameliorate gut inflammatory phenomena. Bassaganya-Riera et al. [47], using in vivo models of IBD, provided molecular evidence that conjugated linoleic acid reduces colitis inflammation employing a PPAR γ-dependent mechanism.

More recently, it was shown that the synthesized jasmonate analog J11-Cl (2-hydroxyethyl 5-chloro-4,5-didehydrojasmonate), structurally similar to cyclopentenone prostaglandin 15d-PGJ_2_, increases PPARγ activity and exerts anti-inflammatory effects, determining a less severe form of intestinal inflammation in dextran sodium sulfate (DSS)-induced colitis in mice. The treatment reduced pro-inflammatory cytokines and chemokines and increased anti-inflammatory cytokines and growth factors [48]. Another research study also supports the role of PPARγ in the amelioration of inflammatory bowel disease. In trinitrobenzene sulfonic acid (TNBS)-induced colitis mice, the andrographolide-lipoic acid conjugate (AL-1) administration alleviates inflammation through inhibiting the expression of TNF-α, IL-1β, and IL-6 and down-regulating the expression of p65 and p-IκB, key regulators of NF-κB pathway. Moreover, COX-2 levels, which are regulated by NF-κB, were reported to control levels, and the expression of PPARγ was increased in AL-1 treated groups [49]. The modulation of PPARγ/NF-κB cascade in intestinal inflammation is related to p21-activated kinase 1 (PAK1), the results of which overexpressed and activated in IBDs [50]. In particular, TNF-α is responsible for the translocation and co-localization of p-PAK1 and p-65 in the nucleus. These events determine the transcriptional activation of NF-κB. Activated PAK1 blocks PPARγ, increasing accumulation of p-65.

TNF-α stimulation can also induce the expression of COX-2, whose transcription can be regulated by several factors such as NF-κB and PPARs [51]. It has been demonstrated that this pro-inflammatory enzyme is induced in the human inflamed large intestine and IL-10 deficient mouse model of IBD [52,53]. COX-2 metabolizes free arachidonic acid (AA) into prostanoids, such as prostaglandins (PGs) and thromboxanes (TXs). The by-products cyclopentenone prostanoids, including 15-deoxy-12,13-didehydro-14,15-didehydro-PGJ_2_ (15d-Δ^12,14^-PGJ_2_), 12,13-didehydro-PGJ_2_ (Δ^12^-PGJ_2_), and PGA_2,_ are ligands of PPARγ, suggesting an interaction between this receptor and COX-2 during inflammation [54,55]. Prostaglandins exert anti-inflammatory effects by inhibiting NF-κB mediated by the blockage of IkappaB kinase and activation of PPARγ [56].

Other evidence suggested the role of the cannabinoid system in IBD, since cannabinoid receptors 1 and 2 (CB1 and CB2) are increased in IBD colonic tissue [57]. Moreover, a strong increase of endocannabinoids (especially anandamide) was found in biopsies from patients with untreated UC [58]. The results of several studies support the hypothesis of a cross-talk between PPARγ and the cannabinoid system in reducing inflammation. Liu et al. indicated PPARγ as a molecular target for synthetic cannabinoids, demonstrating its possible use in various treatments [59]. Furthermore, the sesquiterpene β-caryophyllene (BCP) can reduce DSS-induced colitis in mice with a mechanism associated with CB2 and PPARγ, which leads to the inhibition of pro-inflammatory cytokines and NF-κB [57].

## 3. Perturbation of Gut Microbiota Homeostasis: From Environmental Risk Factors to PPARγ Activation

The gut microbiota has an essential role in preserving intestinal homeostasis, but it is sensible to environmental pollutants and external factors. The microbial community consists of at least 10^11^–10^12^ bacterial cells per millimeter, and the principal phyla include Firmicutes, Bacteroidetes, Actinobacteria, Proteobacteria, Fusobacteria, and Verrucomicrobia [60].

**Figure 2:**
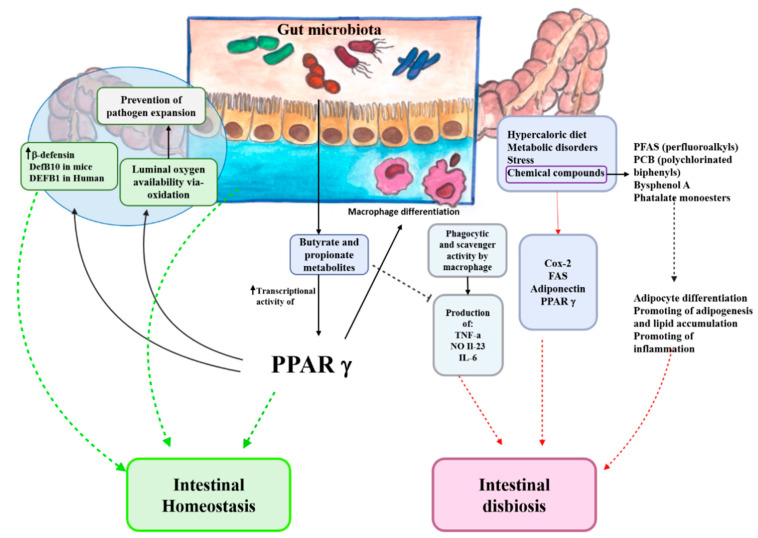
Synergistic action supported by the gut microbiota and PPARγ in the maintenance of intestinal homeostasis. The microbial community produces short-chain fatty acids (SCFAs), such as butyric and propionic acid, which increase the transcriptional activity of intestinal PPARγ, leading to a higher expression of β-defensins (DEFB1 in human and DEFB10 in mice) in the colon. Moreover, PPARγ drives the energy metabolism to β-oxidation: the luminal oxygen availability decreases, preventing the expansion of facultative anaerobic pathogens. PPARγ is also involved in macrophage activation and function, influencing the phagocytic and scavenging activity. Intestinal dysbiosis is the result of endogen and exogen factors. Among the external factors, hypercaloric diet, stress, metabolic disorders, and exposure to chemical compounds permit a state of low-grade inflammation to be established. The reasons are to be found in the altered expression of PPARγ and other inflammation markers. Several chemical compounds may determine alteration in adipose tissue function. Since adipose tissue contributes to intestinal homeostasis, the impairment of PPARγ activity may indirectly promote the onset of intestinal disorders.

PPARγ participates in the maintenance of innate antimicrobial immunity, regulating the expression of a subset of β-defensins in the colon, such as mDefB10 (mice) and DEFB1 (humans) [61]. In particular, a defective killing of Bacteroides fragilis, Enterococcus faecalis, and Candida albicans was observed in Pparγ+/− mice, supporting a PPARγ antimicrobial activity. Regarding the microbiota, alterations in gut-associated microbial community could determine dysbiosis and the onset of inflammatory diseases. It was found that the PPARγ signaling influences the luminal bioavailability of oxygen via β-oxidation, preventing the expansion of pathogenic Escherichia and Salmonella [62]. This observation also suggests the implication of PPARγ in the maintenance of gut microbial health (Figure 2).

Moreover, bacteria can generate metabolites that could interfere with PPAR dependent pathways. In support of this evidence, some studies showed that microbiota metabolites butyrate and propionate increase PPARγ transcriptional activity in HT-29 cells [63]. In IBD patients, the altered microbiota determines dysbiosis with a decrease in butyrate bacteria. The absence of butyrate is critical because it has the anti-inflammatory ability by promoting IL-10 secretion and inhibiting HDACs/NF-κB pathway and increasing PPARγ activity [63].

The treatment of Caco-2 cells with prebiotic oligosaccharides has been shown to induce PPARγ, leading to an anti-inflammatory activity [64]. The beneficial role of prebiotics can be justified by considering that only a healthy gut microbiota is useful in protecting against intestinal inflammation. In light of this, many therapeutic strategies are aimed at restoring the bacterial community. Probiotics and prebiotics like Lactobacillus, Bifidobacterium, Lactulose, Lactosucrose, inulin, oligofructose are just some of these “pharmabiotics” [65].

It has been demonstrated that the proportions of microorganisms may vary according to body fat in humans so that weight loss and weight gain influence the composition of intestinal biota [66]. Other factors contribute to these modifications: diet, metabolic disorders, stress, and chemical compounds. In particular, gut bacteria may interact with chemicals in several ways. First of all, microbes may directly metabolize synthetic molecules ingested with food, or metabolites released by the liver into the bile which then pass into the intestinal lumen. However, these substances can induce dysbiosis interfering with bacterial enzymatic activity [67].

Additionally, in the obesity condition, the microbiota is compromised by the hypercaloric diet. This condition leads to an impairment of the epithelial barrier, which determines an increase in its permeability. Gut microbial lipopolysaccharide (LPS) is responsible for metabolic endotoxemia, to which macrophages respond, transforming to M1 phenotype [68]. Macrophages can be classified as inflammatory macrophages (M1), wound-healing (M2), and regulatory macrophages (Mreg) [69]. They differ from each other based on the conditions (cytokines) in which they are rising [70]. The cytokines are fundamental in macrophage polarization; for example, INF-γ and TNF-α contribute to M1 polarization, while IL-4 polarizes M2, and the regulatory macrophage polarization is influenced by many signals, including IgG immune complexes, IL-10, prostaglandins, or apoptotic cells [70].

Interestingly, patients affected by inflammatory bowel diseases, even when they are in clinical and endoscopic remission maintain a “low-grade intestinal inflammation.” The presence of a residual inflammation is familiar not only in IBDs, but also in type 2 diabetes, cardiovascular diseases, and obesity. The link between chronic or residual inflammation and diseases is modulated by macrophages, which are the primary producers of mediators such as IL-1, IL-6, TNF-α, reactive oxygen intermediates [71]. In normal conditions, bone marrow-derived monocytes are recruited to the intestinal mucosa, where they exert pathogen scavenger activity through TLR4 receptor; they also produce anti-inflammatory cytokines, such as IL-10. Macrophages participate in inflammatory response, secreting pro-inflammatory cytokines, including IL-23 and TNF-α.

Macrophages are localized in the subepithelial lamina propria (LP), and they are identified by several markers, such as F4/80, CD64, and CD11b [72]. Moreover, the distinct properties of macrophages include: phagocytic and scavenger activity against bacteria and pathogens, maintenance of gut homeostasis through anti-inflammatory cytokines production, and eliminating commensals [73]. However, the failure in enteric bacterial tolerance may result in chronic inflammation. Pro-inflammatory macrophages produce large amounts of TNF-α, nitric oxide (NO), IL-23, IL-1β, and IL-6, which are part of anti-microbial activity even if they contribute to tissue damage [74].

The macrophage function is influenced by several factors, including dietary nutrients (fatty acids, vitamins, amino acids). In particular, it has been demonstrated that the treatment with n-butyrate causes a reduction of pro-inflammatory mediators (NO, IL-6, IL-12) produced by macrophages [75]. This evidence suggests an important link between gut microbiota and immune responses.

The precursors of intestinal macrophages are monocytes from bone marrow, and they migrate to the intestinal mucosa, recruited by IL-8 and TGF-β [76]. The subsequent polarization to anergic inflammation cells derives from signals, such as the inhibition of NF-κB by TGF-β [77]. Cytokines and chemokines induce down-regulation of receptor TREM-1 (triggering receptor expressed on myeloid cells) on macrophages involved in potent inflammatory responses.

It has been demonstrated that TREM-1 is involved in IBDs. In particular, an increase in macrophages expressing TREM-1 was observed in patients with inflamed mucosa. This condition leads to high levels of pro-inflammatory cytokines and chemokine (IL-6, IL-8, IL-1β, TNF, etc.). The amplification of inflammatory response mediated by TREM-1 exerts an essential role in exacerbating disease and damage in the mucosa layer [78]. Thus, IBDs and the persistence of signs and symptoms seem to be related to inappropriate macrophage responses, which determine the difficulty of eliminating bacteria. Macrophages also express PPARγ, and many studies are focused on the contribution to this receptor in macrophage differentiation [79], even if other groups define it as not being necessary in this process [80]. Heming et al. showed that the absence of PPARγ in macrophages determines alterations in differentiation pathways and influences the activity of phagocytosis and migration. Moreover, an increase in PPARγ expression can be observed after treatments with glucocorticoids (GC). They also demonstrated that macrophage migration is negatively controlled by PPARγ [81]. These observations suggest that the differentiation and macrophages activity, which are implicated in chronic inflammation, can be modulated by ligands of PPARγ.

### Chemical Compounds and PPARγ Activity

Chronic inflammation is a key aspect not only in CD and UC but also in metabolic syndromes. The activation of inflammatory responses may be the result of the interaction between chemical compounds and PPARγ. In particular, a class of substances named “obesogens” can influence PPARγ and indirectly the homeostasis of adipose tissue. These substances determine a greater susceptibility to the onset of bowel disease, promoting inflammation and dysregulation of metabolic processes. Thus, a correlation between environmental factors and IBDs can be supposed.

Adipose tissue is no longer considered mere storage of fat, but it has an active role in the synthesis and secretion of several hormones [82]. It also participates in bioactive peptide production, such as adipokines and several cytokines [83], produced by adipocytes and preadipocytes.

The importance of adipose tissue’s adequate functioning is reflected by the fact that every perturbation of tissue homeostasis has a systemic impact. Many genetic and environmental factors may determine the adipose tissue’s remodeling, which is demonstrated to be highly dynamic. Two events may derive from these alterations: hyperplasia and hypertrophy of adipocytes [84].

In particular, adipocyte hypertrophy is typical in the obesity state, and it determines the increase in necrotic cell deaths, leading to activation in inflammatory responses and tissue dysfunction [85]. In fact, levels of pro-inflammatory cytokines, such as TNF-α, IL-6, and IL-8 become high, along with the presence of chemoattractant molecules, including MCP-1, which promotes the activity of macrophages and T-cells [86]. Thus, hypertrophic modifications are responsible for harmful consequences, which result in the dysfunction of adipose tissue. The hyperplastic expansion involves the differentiation of pre-adipocytes in adipocytes, where PPARγ and CCAAT/ enhancer-binding protein families play a key role [87]. Hyperplasia leads to an increase in the number of adipocytes without influencing the number of inflammatory cytokines or immune cell recruitment. It represents a protective response against overnutrition [88] since it tries to counteract obesity-associated complications.

The adipose tissue also contributes to the maintenance of intestinal physiology, supporting the local host defenses and participating in immune system responses via adipokines secretion, which are hormone-like factors with auto- and paracrine effects. In particular, leptin and adiponectin were found to exert anti-inflammatory activity by enhancing IL-10 production by anti-CD3-stimulated lamina propria T-lymphocytes (LPL-T) in IBD patients [89]. Nishihara et al. [90] also demonstrated adiponectin’s protective effect against DSS-induced colitis in adiponectin-knockout (APN-KO) mice.

Obesogens influence adipose tissue metabolism in terms of hyperplasia and hypertrophy of adipocytes. They can influence pre-adipocyte differentiation and alter energetic metabolism. Moreover, they can induce inflammation and oxidative stress. Regarding the effects on PPARγ, these substances may increase its expression or bind it, leading to a series of events that culminate with the enhancement of adipogenesis [91].

Many groups of chemical compounds are shown to influence adipose tissue, such as pesticides, fungicides, byproducts of fuel burning such as polycyclic aromatic hydrocarbons (PAHs), components of plastics including bisphenol A (BPA) and its analogues, plasticizing agents used in cosmetics and medicines, or preservatives like parabens.

The anti-inflammatory properties of PPARγ can be modulated by the assumption of several dietary compounds, including glutamine, spicy food, flavonoids [92], or physical activity cardiorespiratory fitness [93]. However, the modulation of this receptor can be related to a large number of external factors. The necessity of deepening this relationship comes from identifying the environmental contribution to the onset of IBDs to implement prevention measures.

It is well-known that many chemicals and by-products of industrial activities can interfere with air, soil, and water quality, influencing the development of living creatures. The concern about these substances determines greater attention and awareness of a healthy lifestyle.

For example, people are exposed to endocrine-disrupting chemicals (EDCs), which are found in everyday products, such as plastic bottles, containers, food, cosmetics, pesticides, etc. EDCs include phthalates, phytoestrogens, triclosan dioxins, parabens, per- and polyfluoroalkyl substances (PFAS), and polychlorinated biphenyls (PCBs). They influence hormonal homeostasis leading to a dysregulation of metabolic responses, including lipid metabolism and adipogenesis [94]. Several studies show a correlation between the activity of these substances and metabolic disruption [95]. Many studies confirmed the activation of PPARγ by chemical compounds. Hurst and Waxman [96] demonstrated the activation of human and mouse PPARγ by phthalate monoesters with an increase in adipocyte differentiation.

Bisphenol A and its derivatives, used in plastic bottles, paper, and other daily products, showed an affinity to human PPARγ because of their structural similarity with 17β-estradiol [97].

The organotin compound tributyltin (TBT), used as biocide and fungicide, binds to PPARγ, promoting adipogenesis and lipid accumulation [98].

PPARγ is a master regulator of lipid homeostasis and adipogenesis, and it also has an essential role in adipocyte differentiation. This concept suggests that any alterations or changes in PPAR-γ-mediated pathways could determine alterations in adipose tissue functions, promoting the onset of intestinal disease indirectly.

Limited experimental and epidemiological data suggest a direct relationship between environmental pollutants or contaminants and IBDs, and there is no clear evidence of the PPARγ involvement. However, this does not mean that external factors could not affect intestinal health. Only a few data are focused on the exposure to air pollution and the risk of IBDs [99] or the role of PFAS contained in drinking water and ulcerative colitis [100]. Still, there is no evidence of a direct connection between these (and other) chemicals and PPARγ.

Probably and until proven otherwise, the environmental factors and pollutants are not directly correlated to IBDs, but they would contribute to enhancing the individual susceptibility to chronic disorders.

Environmental chemicals can also have a role in epigenetic modifications. Several studies were conducted to investigate the ability of air pollutants, endocrine-disrupting compounds, or metals to influence the genome in terms of methylation or histone modifications [101].

For example, maternal exposure to Bisphenol A in rat models results in DNA methylation of specific loci (*A^vy^ and* Capb^IAP^) in fetal epigenome [102].

The strict relation between chemical compounds PPARγ and IBD is still unclear, but the possibility of a PPARγ epigenetic dysregulation is not remote. In fact, in colorectal tumorigenesis, the epigenetic mechanisms would imply changes in PPARγ promoter methylation [103].

Many compounds interfere with PPARγ signaling pathways at different levels. They can act as risk factors: hence the need to get new information by suitable experimental in vivo IBD models.

## 4. Current Experimental Models of IBDs

**Figure 3:**
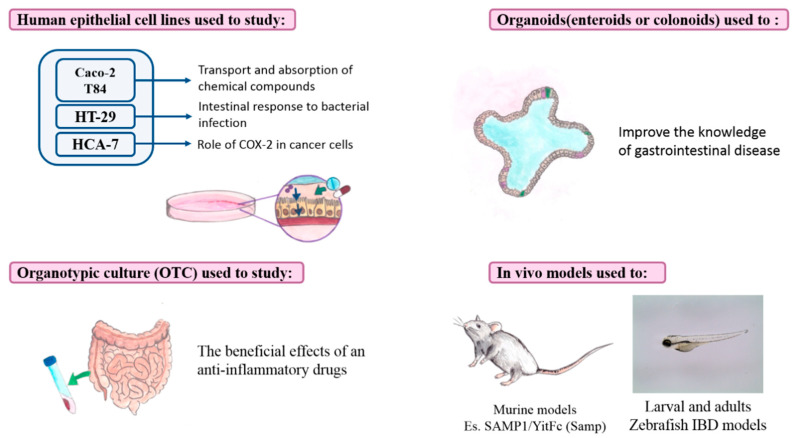
Traditional and innovative in vitro and in vivo models of inflammatory bowel diseases (IBDs). The human epithelial cell lines principally used include: Caco-2, T-84, Ht-29, and HCA-7. They allow one to investigate the function of epithelial barrier in terms of transport and absorption of chemical compounds, and defense mechanisms against bacterial infections. Furthermore, the inflammatory responses in cancer cells may be evaluated. Organotypic culture gives some advantages in pharmacological studies, while organoids offer a more complete representation of intestinal structures with its three-dimensional characteristics. Among the in vivo models, mice are the most used and IBD-like inflammation can be easily induced by means of the exposition to chemical compounds, or using transgenic lines. Future directions may include the use of larval and adult zebrafish, which represent a useful alternative since the costs of management are lower and they allow an easier genetic manipulation and application of imaging techniques.

Tissue and cell culture methodologies significantly contributed to a better comprehension of the underlying mechanisms of IBDs (Figure 3). Among the human epithelial cell lines that have been used for years, there are T84, Caco-2, HT-29, HCA-7. Caco-2 and T84 are typically used as in vitro model systems of the epithelial barrier. In particular, Caco-2 cells allowed the study of the mechanism of transport and the absorption of chemical compounds through the epithelial barrier [104]. HT-29 cells are used to study the intestinal response to bacterial infection and the microbial interactions with the intestinal epithelium [105]. HCA-7 is useful for studying the role of COX-2 in cancer cells and the colorectal epithelial cell polarity [106].

It is well-known that the use of monolayer has some disadvantages related to the limits of bidimensional models. However, a major grade of complexity is reached by making use of co-cultures. Leonard et al. [107] designed a specific in vitro setup, consisting of Caco-2 cells, macrophages, and dendritic cells. This model determines the possibility of studying the mechanism of inflammation, typically present in IBDs. It overcomes the difficulties linked to the use of a monolayer, giving a whole point of view of inflammatory disorders.

Despite the disadvantages, the use of human cancer cell lines represents the starting point for the evaluation of several aspects in IBDs. First of all, the study of molecular processes requires a simple and standardized model. For example, the role of pro-inflammatory mediators on cells or main cellular modifications can be appreciated only by minimizing external fluctuations. Second, pharmacological studies and, in particular, the interaction of drugs with specific receptors cannot be understood in complex models, where many variables could influence the results. However, once these studies are completed, the research can focus on tridimensional models in order to expand knowledge and investigate other aspects.

On the contrary, the organotypic cultures (OTC) allow filling the gap between the extreme simplicity of bidimensional cultures and the complexity of animal models. They are also used to model intestinal inflammation because it is possible to consider the role and the influence of the microenvironmental factors for a better in vitro simulation of physiological conditions. Specimens from patients are usually collected during colonoscopy, and the slices of tissue are transferred into appropriate culture conditions [108]. The applications of OTC are various and include the examination of the effects of therapeutic treatments. For example, biopsies from celiac disease patients have been used to investigate the beneficial effect of anti-inflammatory drugs used in IBD treatment. This study demonstrated that mesalazine induces PPARγ expression, inhibiting oxidative and nitrative species’ production on OTC [109].

However, new modelling strategies have been developed to improve the knowledge of gastrointestinal diseases. One experimental set-up consists of the embedding of human colonic crypts and intestinal stem cells in Matrigel; these cells form enterospheres; i.e., spherical structures with a lumen. After several days of isolation, the organization becomes more complex with the expansion in enteroids and colonoids, miming the structure of epithelium and crypts [110].

Regarding in vivo models, they can be classified based on how the intestinal inflammation is induced.

Dextran sulfate sodium (DSS) and trinitrobenzene sulfonic acid (TNBS) are used to induce colitis in animal (mouse) models. The advantages are mainly related to technical simplicity, and wild-type mice can be used. They allow studying innate immune mechanisms, epithelial injury, and the interaction with adaptative systems. However, neither can be considered a complete representation of colitis in humans [111]. Other models have been developed, such as the IL-10 knockout and the Mdr1a knockout models. They have particular translational relevance, especially in the study of IL-10 receptor polymorphisms involved in early childhood ulcerative colitis and MDR1 polymorphisms, which are associated with human ulcerative colitis [112]. T-cell transfer-induced is model of intestinal fibrosis, permitting the study of immune regulation, Treg, and integrins [113].

SAMP1/YitFc (Samp) mice represent a suitable model to study the underlined mechanisms of CD, since the onset of inflammation is spontaneous and localized in the ileum [111]. Moreover, these mice share features with human Crohn’s diseases: in particular, PPARγ has been identified as a susceptible gene involved in the disease’s onset both in SAMP/Yit mouse and human [43].

Although several models have been developed to gain insights into mechanisms of inflammation and lesions in IBDs, it is clear that the information is incomplete because of the impossibility of having all the same characteristics of the disease in one model. Therefore, the knowledge of inflammation disorders derives from the study of multiple different models. Moreover, the murine models’ limitations are related to difficulties of genetic manipulation, use of imaging techniques, and high maintenance costs.

Given that several gut functions and immune genes are conserved between zebrafish and mammals, the zebrafish is an interesting model organism to investigate fundamental processes underlying intestinal inflammation and injury [114,115].

The advantages of using adult or larval zebrafish as a model for IBDs derive from their similarities with humans in terms of genes involved in intestinal disorders, immune system functions, and anatomical structure, even if Paneth cells and crypts were not detected along with the absence of stomach. Protrusions are called folds, resembling villi, whose size decreases from anterior to posterior part [116]. The intestinal epithelium consists of three types of differentiated cells: mucin-producing goblet cells, absorptive enterocytes, and endocrine cells in the anterior intestine. The submucosa seems not to be present in zebrafish. The smooth muscle layer is directly attached to the mucosa, even if it has a minor complexity. Lamina propria is similar to mammalian connective tissue, despite its simplicity [117]. Although this suggests that the zebrafish digestive system is less complex, the intestine maintains the same functions and organization as the mammal intestines. The rostral, the mid, and the caudal portions are analogs of mammalian small and large intestines [118]. During the developmental stages, the growth and the formation of a complete digestive tract can also be directly followed because of the optical transparency of embryos. Even the immune responses can be traced by means of live imaging techniques.

The zebrafish model allows the study of the innate and adaptative immunity based on the embryos’ developmental stage. In fact, a functional adaptive response is absent during the first days of development, while the innate immunity plays the main role [119].

The larval gut uses antimicrobial peptides (AMPs) produced by epithelial cells as a defense tool against Gram-negative, Gram-positive, fungi, or bacteria [120]. Zebrafish has defensin-like genes, predominantly expressed in the mid intestine [121].

Chemical induced models are adapted from murine models, but they involve the immersion of zebrafish larvae in a specific chemical to permit intestinal exposure. For example, the exposure to TNBS determined a disruption of intestinal architecture and an imbalance of Protobacteria proportion and Firmicutes, which are correlated to enterocolitis severity [122]. It was demonstrated that the morphological and functional changes in zebrafish gut exposed to TNBS are not related to the chemical induction itself, but they are a consequence of the activation of inflammatory responses. Fleming et al. observed significant changes in goblet cell number and TNF-α levels. The model showed similarity with human Crohn’s disease, especially for enlarged lysosomes toward the lumen of the gut’s epithelial lining [123]. The composition of intestinal infiltrate was affected by the intestinal microbiota in adult zebrafish with oxalazone-induced colitis. In particular, variation in bacterial colonies could determine the severity of intestinal damage [124].

The assessment of these models can be done with the administration of anti-inflammatory drugs or antibiotics and the analysis of inflammation and intestinal function. The study of inflammation responses has also been performed by generating transgenic zebrafish with Green Fluorescent Protein expression in neutrophils. Renshaw et al. used this approach to overcome limitations related to histochemical technique so that a single larva could be analyzed using in vivo live imaging [125]. Interestingly, zebrafish, like mice, may be a more robust animal model to study host and microbe’s interaction. Experiments in zebrafish demonstrated that the microbiota increased fat storage in adipose tissue [126] and stimulated fatty acid uptake in the intestinal epithelium and liver [127] suggesting that diet-induced alterations of microbiota could lead to IBD by disturbing the host energy balance. Moreover, studies in experimental animals have indicated that the intestinal microbiota plays a critical role in intestinal inflammation’s pathogenesis [122,128,129].

The possibility of realizing chemical-induced models can be useful to determine the role of such environmental pollutants in the onset of inflammatory bowel disease. Moreover, the involvement of PPARγ during the inflammatory process can be investigated.

A single PPARγ ortholog has been identified in zebrafish, and it shows a high percentage of similarity with the human gene [130]. It has been demonstrated that PPARγ has an important role during early embryonic development; in particular, its early expression around the pancreas and swimming bladder area confirms that it is involved in lipid metabolism since these tissues are the first regions where adipocytes develop [130]. Alterations in zebrafish lipid metabolism showed common features with mammalians and even the presence of PPARγ [131].

This suggests that the interference on lipid homeostasis mediated by a chemical compound could affect PPARγ mediated pathways. Having examined the implications of this receptor in inflammation and the onset of IBDs, it can be postulated that every ligand could act as a risk factor.

For example, several studies showed that parabens, classified as endocrine disruptors, can interfere with adipocyte differentiation, promoting adipogenesis and modulating the expression of adipokines, adiponectin, and leptin [132]. The effects of these substances have been investigated in zebrafish, and the observations indicated that propylparaben interferes with lipid metabolism [133].

Other chemicals were the subject of studies, such as BPA [134], perfluorooctane sulfonate, and tributyltin [135]. Nevertheless, there is no clear evidence of the association between endocrine disruptors and the onset of IBDs in zebrafish. Furthermore, this lack of information determines the need for further investigations utilizing the design of a new kind of zebrafish IBD model.

## 5. Conclusions

The PPARs family is involved in inflammation responses associated with IBDs. Several studies have demonstrated the role of PPARγ in dysregulation of gut homeostasis and how it determines the characteristic features of the diseases. A better knowledge of the underlined mechanisms is essential for developing targeted therapeutic strategies, which represents the gold standard in personalized medicine. Until now, in vitro and in vivo models could not wholly satisfy this need since the onset of IBDs results from a mixture of risk factors, genetic susceptibility, food habits, dysregulation of immune responses, etc.

However, these hypotheses taken together could not totally explain the increase in terms of incidence and prevalence of IBDs worldwide.

Thus, there may be the contribution of external/environmental factors, which could increase individual susceptibility. Since the correlation between pollutants and IBDs has not been examined in-depth, it is necessary to define a new experimental setup.

Murine models are typically used to investigate etiopathogenesis of inflammation disorders, but they have limitations in terms of costs and techniques.

In this scenario, zebrafish represent the possibility of filling the lack of knowledge representing a suitable model. It shares the main structural features of digestive systems or mechanism of inflammation and represents a simplified means of disserting the complex background of IBDs. PPARγ is well conserved in vertebrates and other isoforms, so the zebrafish model will be a useful tool to study the correlation between external factors and the activation of the γ-isoform dependent pathway.
